# L-Arginine Reduces Nitro-Oxidative Stress in Cultured Cells with Mitochondrial Deficiency

**DOI:** 10.3390/nu13020534

**Published:** 2021-02-06

**Authors:** Camila D. S. Barros, Jomênica B. Livramento, Margaret G. Mouro, Elisa Mieko Suemitsu Higa, Carlos T. Moraes, Celia Harumi Tengan

**Affiliations:** 1 Department of Neurology and Neurosurgery, Escola Paulista de Medicina, Universidade Federal de São Paulo, Sao Paulo 04039-032, Brazil; dantas.ca@hotmail.com (C.D.S.B.); jomenica@gmail.com (J.B.L.); 2 Emergency and Nephrology Division, Escola Paulista de Medicina, Universidade Federal de Sao Paulo, Sao Paulo 04039-032, Brazil; gori@nefro.epm.br (M.G.M.); emshiga@unifesp.br (E.M.S.H.); 3 Department of Neurology, University of Miami Miller School of Medicine, Miami, FL 33136, USA; CMoraes@med.miami.edu

**Keywords:** arginine, mitochondrial disease, nitric oxide, oxidative stress, nitration, mitochondrial DNA

## Abstract

L-Arginine (L-ARG) supplementation has been suggested as a therapeutic option in several diseases, including Mitochondrial Encephalomyopathy, Lactic Acidosis, and Stroke-like syndrome (MELAS), arguably the most common mitochondrial disease. It is suggested that L-ARG, a nitric oxide (NO) precursor, can restore NO levels in blood vessels, improving cerebral blood flow. However, NO also participates in mitochondrial processes, such as mitochondrial biogenesis, the regulation of the respiratory chain, and oxidative stress. This study investigated the effects of L-ARG on mitochondrial function, nitric oxide synthesis, and nitro-oxidative stress in cell lines harboring the MELAS mitochondrial DNA (mtDNA) mutation (m.3243A>G). We evaluated mitochondrial enzyme activity, mitochondrial mass, NO concentration, and nitro-oxidative stress. Our results showed that m.3243A>G cells had increased NO levels and protein nitration at basal conditions. Treatment with L-ARG did not affect the mitochondrial function and mass but reduced the intracellular NO concentration and nitrated proteins in m.3243A>G cells. The same treatment led to opposite effects in control cells. In conclusion, we showed that the main effect of L-ARG was on protein nitration. Lowering protein nitration is probably involved in the mechanism related to L-ARG supplementation benefits in MELAS patients.

## 1. Introduction

L-Arginine (L-ARG) supplementation has been considered as a therapeutic option in different conditions, such as immunological dysfunctions, cardiovascular diseases [1], obesity [2], diabetes [3,4], and mitochondrial diseases [5,6]. Its main effect is related to NO production, as L-ARG is a substrate for nitric oxide synthase.

Good clinical responses were observed after L-ARG supplementation in patients with MELAS (mitochondrial encephalomyopathy, lactic acidosis, stroke-like episodes), a mitochondrial disease with stroke-like episodes as the most typical manifestation [5]. This common syndrome among patients with mitochondrial diseases is frequently caused by a mitochondrial DNA (mtDNA) mutation (m.3243A>G) in the tRNA^Leu(UUR)^ gene and is associated with oxidative phosphorylation (OXPHOS) deficiency and decreased ATP production [7].

The mechanisms involved in the L-ARG-induced clinical improvement in MELAS patients are not clear. It is hypothesized that L-ARG can increase NO synthesis, promoting vasodilation due to NO action in vascular smooth muscle [8,9,10]. This hypothesis is supported by results showing the improvement of microvascular cerebral flux and reduction in tissue damage caused by stroke-like episodes in these patients [11]. L-ARG supplementation is also supported by the finding of L-ARG deficiency in these patients [8,12].

L-ARG is a semi-essential amino acid obtained by endogenous synthesis or by exogenous sources [13,14]. In mammalian cells, L-ARG is metabolized by several enzymes such as nitric oxide synthesis (NOS), arginase, arginine decarboxylase (ADC), and arginine:glycine amidinotransferase (AGAT). L-ARG metabolism produces different metabolites: NO, urea, L-ornithine, L-citrulline, creatine, agmatine, glutamate, proline, and polyamines [15,16,17]. NOS synthesizes NO with L-ARG as a substrate by one of three NOS isoforms: neuronal NOS (nNOS), inducible NOS (iNOS), and endothelial NOS (eNOS). nNOS and eNOS are regulated by calcium-calmodulin, whereas iNOS is calcium-independent [13,18,19].

NO has different functions depending on the type of tissue and cell involved. In blood vessels, NO acts as a vasodilator, whereas in the central nervous system NO is a neurotransmitter and mediates immunological responses [13,20]. L-ARG supplementation increases NO, improving perfusion in microvascular compartments, including skeletal muscle. Thus, it was also proposed that L-ARG benefits could include improvement in other clinical manifestations in MELAS, such as muscle weakness, exercise intolerance, and diabetes [8]. In addition, NO has specific effects in mitochondria, such as the respiratory chain regulation, through the inhibition of Complex IV. It is also involved in the mitochondrial biogenesis signaling pathway and the generation of reactive oxygen and nitrogen species as an inhibitor of specific sites in the respiratory chain. Although the leading hypothesis to explain L-ARG action is the improvement of cerebral blood flow due to an increase in NO synthesis, the exact mechanisms are still unclear. We hypothesize that L-ARG effects could be related to the improvement of mitochondrial function or interfere in the oxidative status due to reactive nitrogen species. Thus, our aim was to evaluate the effects of L-ARG in cells with mitochondrial deficiency due to m.3243A>G, focusing on mitochondrial function, NO synthesis, and nitro-oxidative stress.

## 2. Materials and Methods

### 2.1. Cell Culture

Cell culture was performed with cybrid cells derived from osteosarcoma line 143B (TK^−^). Cybrids cells containing the m.3243A>G were obtained by fusing enucleated cells from patients with this mutation with cells of an immortal lineage. The construction and characterization of these cybrid cells have previously been described [21]. Cybrids homoplasmic for the m.3243A>G mutation and the wild-type mtDNA (143B) were cultured at 37 °C with 5% CO_2_ in Dulbecco’s Modified Eagle Medium (DMEM) with D-glucose (4.5 g/L), 44 mM of sodium bicarbonate, non-essential amino acids (100 μg/mL; including 0.4 mM L-ARG hydrochloride), 1× vitamin solution (Gibco BRL, Grand Island, NY, USA), 1 mM of sodium pyruvate, penicillin (100 U/mL), streptomycin (100 μg/mL), amphotericin (0.5 μg/mL), 10% fetal bovine serum (FBS), and uridine (100 μg/mL) in deficient cells. Cells were treated with 1 mM of L-ARG [5] and a combination of L-ARG and 1 mM of L-NMMA (NOS antagonist) [22] for 24 h in DMEM with no FBS. An additional treatment with 1 mM of L-NMMA was included only for the determination of intracellular NO. The mutation load in m.3243A>G cells was higher than 98% and did not change after treatment.

### 2.2. Respiratory Chain Enzyme Activities

#### 2.2.1. Spectrophometric Assays

Cells were resuspended in mannitol buffer (225 mM mannitol, 75 mM sucrose, 10 mM Tris-HCl, 0.1 mM EDTA, pH 7.2) and homogenized in a Potter-Elvehjem homogenizer. The assays were performed in a Cary^®^ 50 Bio UV-Vis spectrophotometer (Varian, Mulgrave, Victoria, Australia) using the software Kinetics. We determined the activities of citrate synthase (CS), complex II (C-II) and IV (C-IV), according to the protocols described in Barrientos et al. [23].

#### 2.2.2. Cytochemical Staining

Cells were grown on coverslips treated with poly-L-lysine. Cytochemical reactions were performed for cytochrome c oxidase (COX, C-IV) and succinate dehydrogenase (SDH; C-II). COX cytochemistry: cells were incubated in 0.1 M phosphate buffer at pH 7.4, containing 3,3′ diaminobenzidine (1 mg/mL), cytochrome c from horse (1 mg/mL), and catalase (200 μg/mL) at 37 °C for 3 h. SDH cytochemistry: cells were incubated in 0.2 M phosphate buffer, pH 7.2, containing nitroblue tetrazolium (1 mg/mL) and succinic acid (50 mg/mL) at 37 °C for 3 h.

### 2.3. Expression of Complex II Flavoprotein Subunit (Fp)

Fp expression was used as a marker of mitochondrial mass. Cells were homogenized in mannitol buffer with 10% sodium dodecyl sulfate (SDS), 10 mM ethylenediaminetetraacetic acid, 10 mM Ethylene glycol-bis(2-aminoethyl ether)-*N*,*N*,*N*′,*N*′-tetraacetic acid, 63 mM Tris pH 6.8, 6% glycerol, and 50 mM 1,4-dithiothreitol. Samples were heated at 94°C, then run in a 12% acrylamide denaturing gel and transferred to a PVDF membrane. The membrane was incubated with 5% milk in Tris-saline buffer with 0.1% Tween-20 for one hour, followed by incubation with the primary antibody at 4 °C overnight. The incubation with the secondary antibody was performed for one hour at room temperature. The antibodies used were anti-C-II, flavoprotein subunit—Fp (1 μg/μL; Invitrogen, 1:1000 dilution); anti-β actin, (1 μg/mL; ABCAM, 1:500 dilution); anti-rat IgG horseradish peroxidase (HRP) conjugated (0.8 mg/mL; BioRad; 1:50,000 dilution); anti-goat IgG HRP conjugated (400 μg/mL; Santa Cruz, 1:10,000 dilution). The detection was conducted with the ECL^TM^ Western Blotting Analysis System (GE Healthcare, Piscataway, NJ, USA), followed by autoradiography.

### 2.4. Quantification of mtDNA

Genomic DNA was extracted from 143B and m.3243A>G cells with the QIAmp DNA (Qiagen, Germantown, MD, USA) extraction kit. The quantification of the mtDNA copy number was performed by real-time polymerase chain reaction (PCR) according to the protocol described by Venegas et al. [23,24], with a few modifications. Assays included two independent amplifications of mtDNA (*MTL1*) and nuclear DNA (*B2M*). The primer sequences were as follows: (MTL1) 5′-CACCAAGAACAGGGTTTGT-3′ and 5′-TGGCCATGGGTATGTTGTTA-3′, (B2M) 5′-TGCTGTCTCCATGTTTGATGTATCT-3′, and 5′-TCTCTGCTCCCCACCTCTAAGT-3′. Reactions were run on a 7500 Real-Time PCR System (Applied Biosystems) with Power SYBR^TM^ Green PCR Master Mix (Applied Biosystems), 10 ng of DNA, and 0.5 µM of each primer in a 10 µL of total volume. The PCR efficiencies were 94% and 93%, with *MTL1* and *B2M* primers, respectively. The amplification conditions were 1 cycle at 50 °C for 2 min and 95 °C for 2 min, 40 cycles with 95 °C for 15 s and 62 °C for 1 min, followed by 95 °C for 15 s, 50 °C for 1 min, and 95 °C for 15 s.

### 2.5. Detection of Nitrated Proteins

Nitrated proteins were detected by immunostaining with the antibody against 3-nitrotyrosine. Cells were grown on coverslips and incubated with 10% goat serum for 30 min, followed by incubation with the primary antibody (1 mg/mL; Molecular Probes; 1:50 dilution) at 4 °C overnight. Incubation with the secondary antibody (anti-rabbit IgG fluorescein isothiocyanate conjugated 1.5 mg/mL; FITC, Vector Laboratories; 1:200 dilution) was for 1 h, followed by incubation with Hoechst Dye 33,342 (1 µg/mL) for 5 min. The positive control was obtained with cells incubated with 1 mM of NaNO_2_ and 1 mM of H_2_O_2_ in acetate buffer pH 5 for 5 min, then fixed with 4% formaldehyde.

Immunostaining images were obtained with a 20× objective, and the fluorescence intensity was quantified using the ImageJ 1.46 software. We analyzed images from different regions on the coverslip. The area of cellular staining was selected using the freehand tool, and the intensity of the delimited area was measured and expressed as optical density. The results were divided by the background intensity (regions with no cells) to correct for any variations in the light intensity. The results were obtained from two independent experiments with a quantification of 10 cells in each experiment. The results were expressed as a percentage of the mean number of untreated cells in each experiment.

### 2.6. Nitric Oxide Synthesis

#### 2.6.1. Quantification of Extracellular NO

Extracellular NO content was evaluated in culture medium and detected by the Nitric Oxide Analyzer (Sievers Instruments, Inc., Boulder, CO, USA). NO was measured after a chemiluminescent reaction between NO and ozone [25]. The total cellular protein content was measured by the bicinchoninic acid protein assay. The NO content was corrected by the total cellular protein in each plate.

#### 2.6.2. Quantification of Intracellular NO

The intracellular NO content was measured using the intracellular NO marker, DAF-FM (4-amino-5-methylamino-2′,7′-difluorofluorescein) diacetate. Cells (3 × 10^4^) were seeded in 12-well plates in DMEM with 10% FBS and no phenol red. We used DMEM with no phenol red in all steps. On the second day, we replaced the medium with DMEM with no FBS. On the third day, the cells received each specific treatment (L-ARG, L-NMMA) for 24 h. After this period, the wells were washed with DMEM and then incubated with 5 μM of DAF-FM in DMEM at 37 °C for 1 h. After incubation, the cells were kept in DMEM for 30 min for the complete de-esterification of the intracellular diacetates. Cells were harvested with TrypLE Express (Gibco, Grand Island, NY, USA) and resuspended in DMEM. The quantification of fluorescent signal was carried out with the Versa Fluor™ Fluorometer (BioRad), at 495 nm (excitation) and 515 (emission). After reading the DAF-FM signals, the cells were incubated with Hoechst dye 33,258 (1 µg/mL, Molecular Probes) and the fluorescence signal was read at 495 nm (excitation) and 515 nm (emission). The DAF fluorescence intensity was corrected to variations in cell number using the Hoechst dye fluorescence.

### 2.7. Statistical Analysis

Values are shown as means ± standard errors. Statistical analyses were performed by two-way ANOVA considering the following factors: cell type (143B and m.3243A>G) and treatment, except in nitrated protein analysis, where we considered the experiment (1 and 2) and treatment for each cell type. The levels of treatment were: (a) no treatment, L-ARG and L-ARG + L-NMMA for enzyme activities and extracellular NO content; (b) no treatment, L-ARG, L-ARG + L-NMMA and L-NMMA for intracellular NO content; (c) no treatment and L-ARG for Western blotting, mtDNA content, and nitrated proteins. Multiple comparisons were performed by the Bonferroni or Tukey’s tests. A *p*-value < 0.05 was considered statistically significant. Statistical analyses and graphs were completed using GraphPad Prism for macOS, version 9.0.0 (GraphPad Software, LLC, San Diego, USA).

## 3. Results

### 3.1. L-ARG Had No Effects on Respiratory Chain Enzyme Activities and Mitochondrial Mass

**Figure 1:**
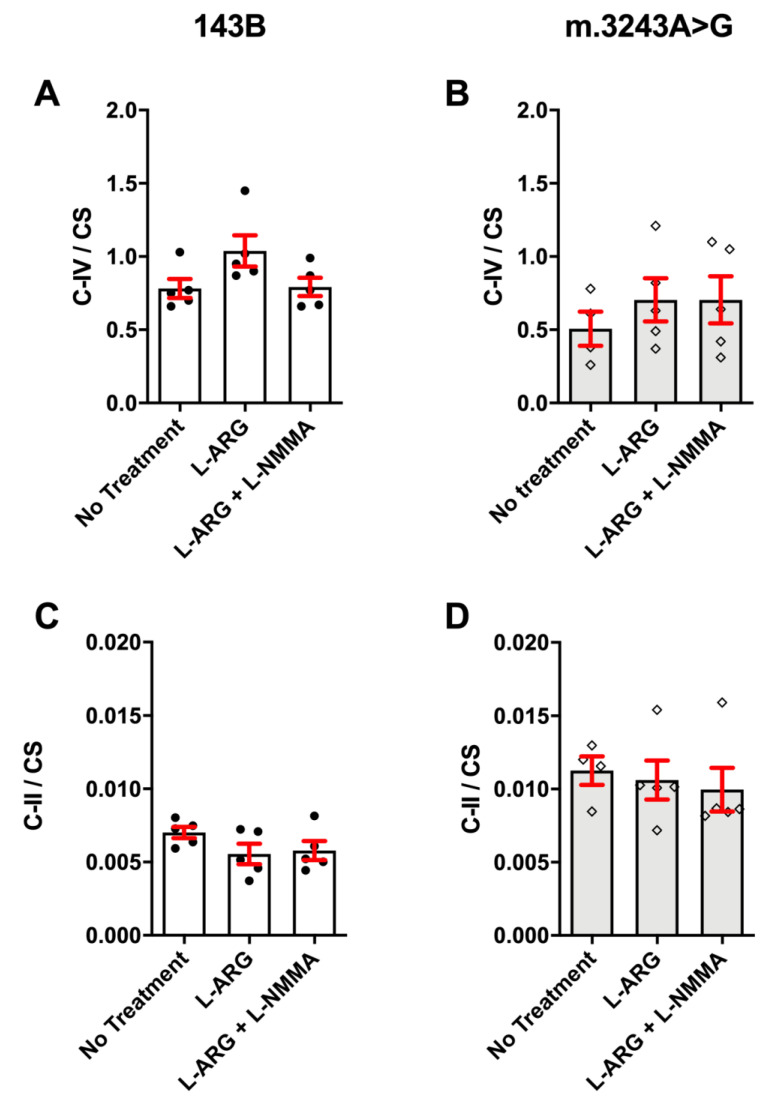
Mitochondrial enzyme activities measured by spectrophotometry. Mitochondrial enzyme activities were corrected to mitochondrial content using Citrate Synthase (CS) in 143B (**A**,**C**) and m.3243A>G (**B**,**D**) cells. No significant differences were found in C-IV and C-II with the three conditions (no treatment, L-ARG and L-ARG + L-NMMA) and in both cell types (m.3243A>G or 143B). Statistical analysis was performed using two-way ANOVA followed by Tukey’s Post Hoc test. C-II = Complex II; C-IV = Complex IV; CS = citrate synthase.

**Figure 2:**
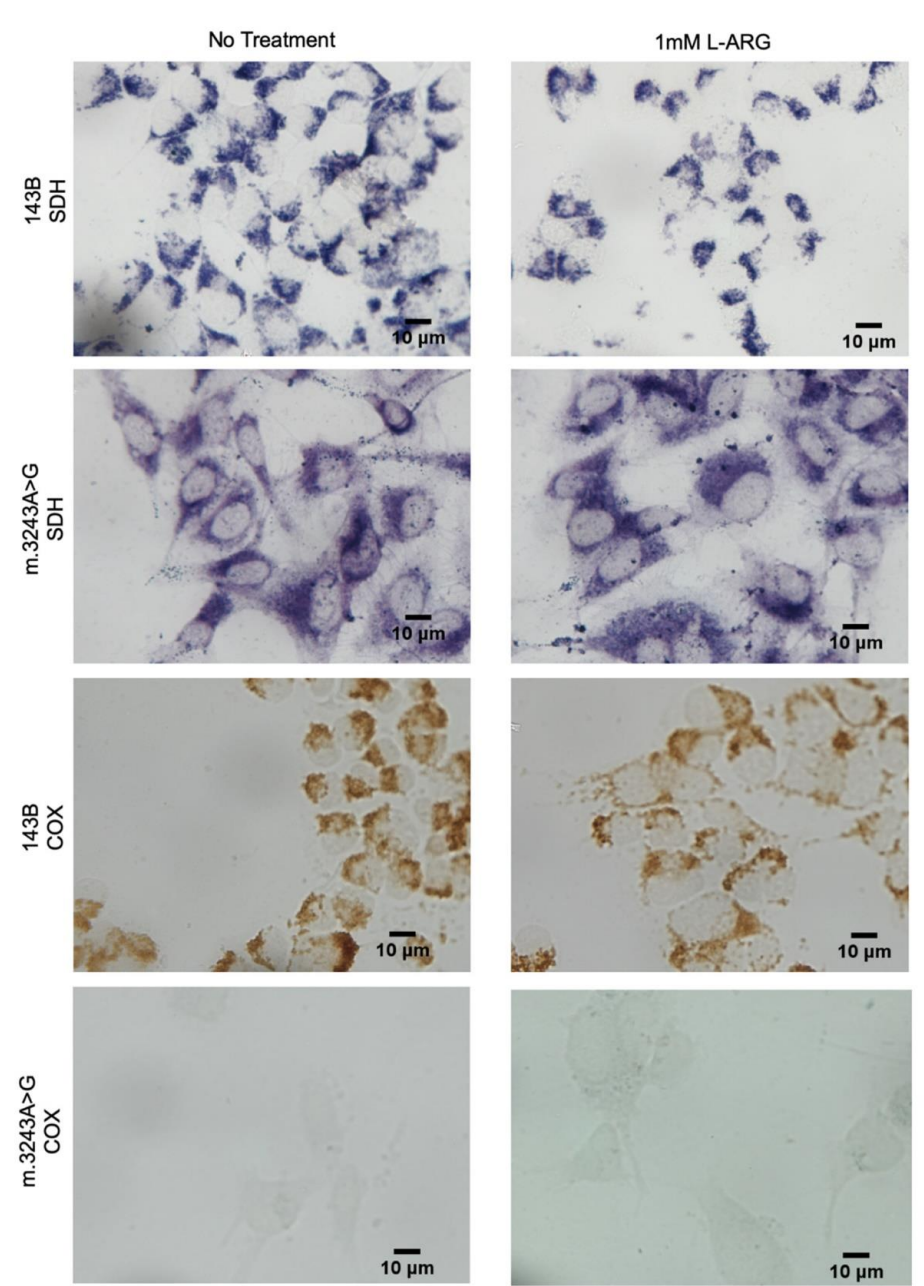
SDH and COX activities by cytochemical staining showed no improvement in COX. 143B cells had preserved SDH and COX activities with and without L-ARG. SDH activity was also preserved in m.3243A>G cells in both conditions, with and without treatment, but had a low COX activity in basal conditions. L-ARG treatment did not modify this pattern of severe COX deficiency, and no cells had COX activity.

To evaluate whether L-ARG supplementation could improve mitochondrial function, we analyzed the respiratory chain enzyme activities, C-II and C-IV. Because C-IV has subunits encoded by the mtDNA, whereas C-II is exclusively coded by the nuclear DNA, we expected to see a defect in COX only [26]. The C-IV activity (C-IV/CS) was 64% lower than that of controls and the C-II was 57% higher, but we did not observe any significant changes with L-ARG treatment in m.3243A>G and 143B cells (Figure 1). We also performed cytochemistry assays to evaluate whether L-ARG could induce the C-IV activity restoration in isolated cells, but even though the C-IV defect was even more evident in the m.3243A>G cells we found no COX activity improvement upon L-ARG treatment (Figure 2).

**Figure 3:**
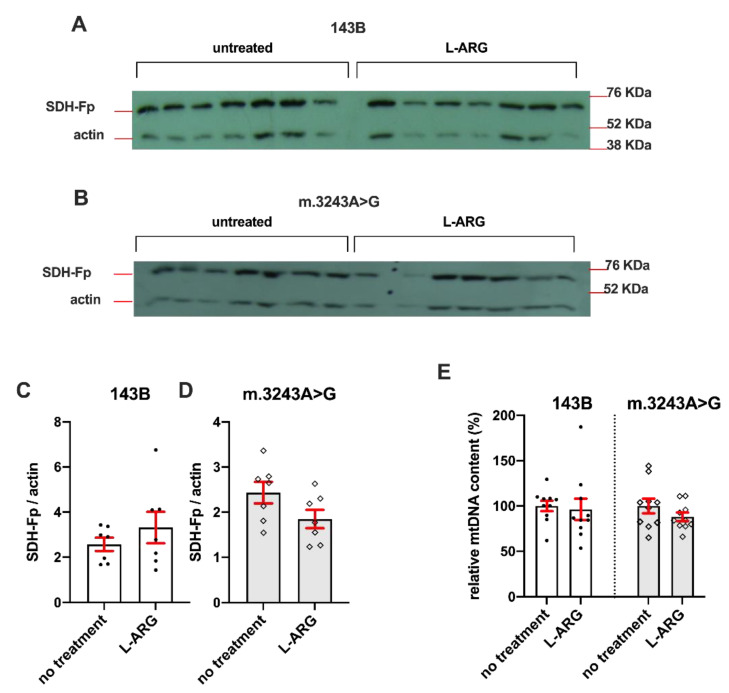
Mitochondrial mass was not altered by L-ARG supplementation. Mitochondrial content was evaluated by Western blotting (**A**–**D**) and mtDNA quantification (**E**). Fp expression, which is not affected by the m.3243A>G mutation, was detected by Western blotting in 143B (**A**) and m.3243A>G cells (**B**). Actin expression was used for the correction of protein content. Quantification of the bands did not demonstrate any statistical difference between L-ARG and untreated cells (**C**,**D**). Relative mtDNA content was expressed as the mtDNA/nDNA ratio relative to each experiment’s mean of the untreated group. The graph (**E**) shows no difference between the untreated and L-ARG group in both cells, 143B and m.3243A>G. Results are from two independent experiments (*n* = 5 in each group). Statistical analysis was conducted using a two-way ANOVA followed by Tukey’s post hoc test.

The mitochondrial mass was evaluated by Western blotting and mtDNA content (Figure 3). The analysis of Fp expression, a C-II subunit, showed no significant differences in cells treated with L-ARG when compared to untreated cells (Figure 3A–D). Additionally, L-ARG did not increase mDNA content in cells treated with L-ARG (Figure 3E).

### 3.2. L-ARG Supplementation Affected NO Synthesis and Protein Nitration

**Figure 4:**
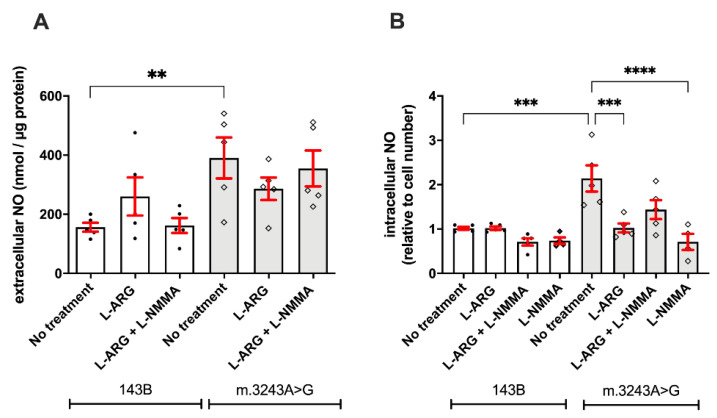
L-ARG and L-NMMA decrease the intracellular NO in MELAS cells. Determination of NO content was conducted in the culture medium of cells treated with L-ARG, L-ARG + L-NMMA. Cells with no treatment served as controls. The basal level of extracellular NO was significantly increased in m.3243A>G (*p* = 0.007, one way ANOVA) when compared to 143B cells (**A**). However, no significant differences were observed after treatment with L-ARG or the combination of L-ARG and L-NMMA in both cell lines. Intracellular NO was quantified using an NO-fluorescent indicator (DAF-FM), corrected by cell number using Hoechst dye. At basal condition, m.3243A>G cells had increased intracellular NO concentration compared to 143B cells (**B**). Treatment with L-ARG or L-NMMA did not change the intracellular NO in 143B cells but decreased the intracellular NO in m.3243A>G cells. L-NMMA significantly decreased the intracellular NO in m.3243A>G cells, suggesting that most NO originated from NOS. Statistical analysis was performed using two-way ANOVA followed by Tukey’s post hoc test. White bars = 143B cells; gray bars = m.3243A>G. ** *p* ≤ 0.01; *** *p* ≤ 0.001; **** *p* ≤ 0.0001.

We evaluated the NO content to verify whether L-ARG supplementation would affect the NOS pathway. We observed that m.3243A>G cells had higher levels of extracellular (390.4 ± 69.09 nmol/µg vs. 156.3 ± 15.07 nmol/µg; *p* = 0.007; Figure 4A) and intracellular NO (1.43 ± 0.03 vs. 0.28 ± 0.03; *p* = 0.008; Figure 4B) when compared to 143B cells. Treatment with L-ARG, L-ARG + L-NMMA, or L-NMMA did not modify the extracellular NO levels in either cell line (Figure 4A). However, we observed clear effects on intracellular NO, with decreases in NO content in m. 3243A>G cells with L-ARG (*p* = 0.03) or L-NMMA (*p* = 0.006) treatments (Figure 4). Differently, 143B cells were only affected by combined treatment with L-ARG and L-NMMA (*p* = 0.04), with lower intracellular NO levels.

**Figure 5:**
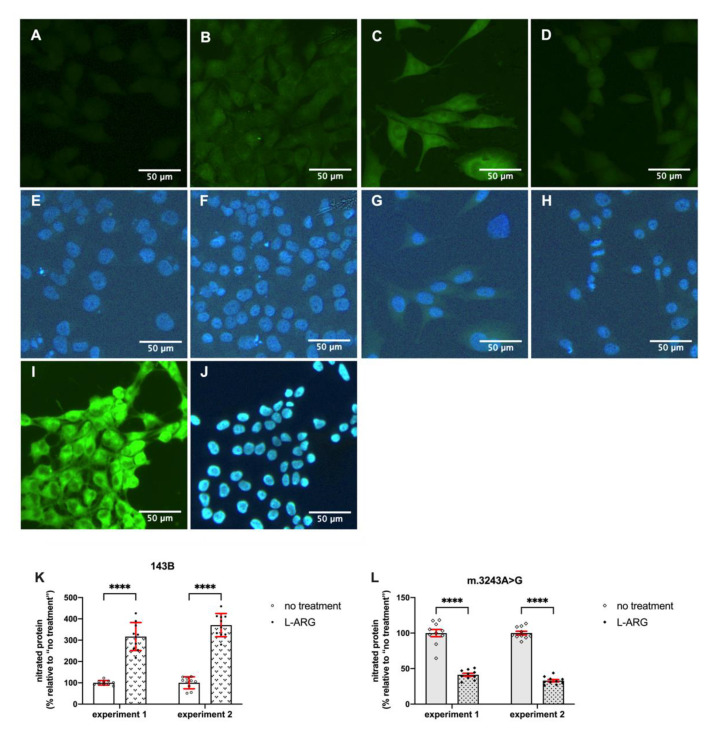
Nitrated proteins by immunostaining showed opposite effects of L-ARG in MELAS and control cells. Immunostaining was performed on 143B (**A**,**B**,**E**,**F**), m.3243A>G cells (**C**,**D**,**G**,**H**) and a positive control (**I**,**J**). Cells received no treatment (**A**, **C**) or L-ARG (**B**,**C**). Nitrated proteins were detected with the anti-3-Nitro-tyrosine antibody (**A**–**D**,**I**) nuclei were stained with Hoechst dye (**E**–**H**,**J**). The panel shows that immunostaining is low in 143B cells (**A**) but increased after L-ARG (**B**). m.3243A>G cells show evident staining (**C**), which was reduced with L-ARG. Immunostaining was quantified by image analysis (**K**,**L**) in two independent experiments. Dots represent the measured immunostaining in each cell and expressed as the percentage of the “no treatment” mean. Open dots are from no treatment and closed dots are from cells treated with L-ARG. A total of ten cells were quantified in each group. In both experiments, the results show an increase in nitrated proteins in 143B cells after L-ARG treatment (**A**). L-ARG induced an opposite result in m.3243A>G cells with a reduction in nitrated proteins (**B**). Statistical analysis was performed using two-way ANOVA followed by Bonferroni’s post hoc test., **** *p* < 0.0001.

The presence of 3-nitro-tyrosine is a marker of nitro-oxidative stress and indicates protein nitration. m.3243A>G showed high-intensity cytoplasmic immunofluorescence for 3-nitro-tyrosine at basal conditions, suggesting nitro-oxidative stress (Figure 5). Positive immunostaining was found in the vast majority of m.3243A>G cells (98.8% in experiment 1, *N* = 339 cells; 98.6% in experiment 2, *N* = 293 cells). Interestingly, and in agreement with our fluorometer results, treatment with L-ARG induced opposite effects in 143B and m.3243A>G cells. L-ARG induced protein nitration in 143B cells, whereas in m.3243A>G cells the 3-nitro-tyrosine immunostaining was decreased compared to untreated cells. The quantification of fluorescence intensity (Figure 5) confirmed that these differences were statistically significant (*p* < 0.0001). Immunoreactivity was homogeneous in L-ARG-treated cells and present in high proportions in 143B (99.4%; *N* = 320 cells in experiment 1; *N* = 309 in experiment 2) and m.3243A>G (97.6% in experiment 1, *N* = 292; 98.8% in experiment 2; *N* = 241).

## 4. Discussion

Due to positive clinical responses in stroke-like episodes, L-ARG supplementation has been recommended to patients with MELAS, especially in the acute phase of the disease [27,28]. The pathogenesis of stroke-like episodes is attributed to the co-existence of mitochondrial cytopathy and mitochondrial angiopathy, causing impairment in oxidative phosphorylation and vasodilation, respectively [28]. However, we still do not know how L-ARG impacts mitochondrial function in these cases. L-ARG is a substrate for NOS, and NO has been involved in multiple mitochondrial processes, including mitochondrial biogenesis [3,14] and respiratory chain regulation [29,30]. Thus, it was expected that L-ARG would affect mitochondrial function or mass in m.3243A>G cells, which was not confirmed by our results.

Despite the severity of the mitochondrial deficiency in m.3243A>G cells, our study demonstrated a robust effect of NO content in protein nitration. First, we confirmed our previous results showing that m.3243A>G cells have an increased intracellular NO level [22]. This result contrasts with that of another study showing decreased NO levels in cybrids derived from neuroblastoma with the same mutation and similar reduction in mitochondrial enzyme activities in homoplasmic cells [5]. Although we do not have an explanation for this, these somehow contradictory results could be related to differences in the method of evaluating NO, the type of cells analyzed, or the nuclear background involved. Desquiret-Dumas et al. evaluated NO on culture supernatant by an indirect method that detects nitrite/nitrate, while we used a method for detecting intracellular NO. On the other hand, Koga et al. found increased NO metabolites in plasma or urine in patients with MELAS during the inter-ictal phase [31]. In the same study, NO metabolites were decreased during the acute phase, suggesting a more complicated process with variations on NO levels depending on the disease state. Interestingly, Koga et al. [30] showed that NO metabolites levels returned to inter-ictal phase concentrations after 24 h of L-ARG supplementation. The presence of protein nitration in m.3243A>G cells also supports the notion that NO is increased. Cells with m.3243A>G have an increased generation of superoxide due to C-I deficiency [7]. Superoxide, in combination with NO, generates peroxynitrite, a very reactive radical that promotes protein nitration. Furthermore, protein nitration was also observed in blood vessels in the skeletal muscle of patients with MELAS [31].

Treatment with L-ARG led to very unexpected responses. We expected that L-ARG would increase NO synthesis because L-ARG is a substrate for NOS. However, we found that L-ARG decreased both NO and protein nitration in m.3243A>G cells. In contrast, an opposite effect was obtained in 143B cells, with the induction of protein nitration by L-ARG, but with no effect on the intracellular NO content. Increased or decreased NO synthesis induced by L-ARG was also found by Xiong et al. [32] but after acute (30 min) or chronic treatment (7 days) of endothelial cells [32]. The expected response with increased NO was found in acute treatment, but the opposite effect, with decreased NO synthesis, was found with chronic treatment [32]. It was proposed that this paradoxical response was due to an uncoupled state of eNOS, which would produce superoxide instead of NO [32]. In this condition, L-ARG would decrease the excessive production of NO in m.3243A>G cells, consequently decreasing protein nitration. Osteosarcoma cells express two NOS isoforms, eNOS and iNOS, but eNOS is the predominant isoform at basal conditions [33].

Thus, we speculate that it is likely that, in our study, the opposite responses could be due to different coupling states, with uncoupled NOS in m.3243A>G, whereas in 143B cells NOS was probably coupled. Thus, in 143B it is possible that NO was still being synthesized but rapidly diverted to nitration, not altering the level of intracellular NO. The basal level of NO may be a key factor in differentiating the coupling state after L-ARG treatment, and the modification of the coupling state of NOS could be one mechanism to prevent critical levels of NO [34].

The mechanisms involved in the uncoupling of NOS have still not been completely elucidated. Some factors can induce NOS uncoupling, such as the decreased availability of BH4 and L-ARG, the accumulation of methyl-arginine (L-NMMA and ADMA), and the S-glutathionylation of eNOS [35]. L-ARG availability can be regulated by the recycling of citruline through the argininosuccinate synthase and argininosuccinate lyase enzymes [6], but it is a pathway that needs further clarification in the osteosarcoma cell lines. There are indications that argininosuccinate synthase is involved in the metastatic process of osteosarcoma [36], but we still do not know if argininosuccinate lyase is expressed in these cells. Arginase is another important factor that regulates the availability of L-ARG. Interestingly, Scalera et al. found that long-term treatment with L-ARG induced decreased NO synthesis, associated with increased arginase activity, reducing L-ARG availability [37]. Arginases have regulatory actions on NO synthesis, modulating L-ARG availability, and are possibly part of a mechanism for limiting NO production [38]. Moretto et al. showed that chronic treatment with L-ARG increased arginase activity in rats. This effect varies in different tissues; for instance, in the lung the activity is increased, while there is no effect in the brain [39]. However, higher arginase activity and uncoupled NOS may lead to an increase in superoxide generation, which argues against the reduction in protein nitration seen in our study. It is possible that this reduction may have occurred due to a predominant decrease in NO over the increase in superoxide.

## 5. Conclusions

In conclusion, our results show that 143B and m.3243A>G cells have different responses to L-ARG treatment concerning NO synthesis and protein nitration. L-ARG reduced protein nitration in m.3243A>G but increased nitration in 143B cells. Our hypothesis to explain these discrepancies is that there are differences in the coupling of NOS and arginase activity, which may be related to the basal NO level. However, additional studies are still necessary to clarify the processes involved in the L-ARG response. Lowering protein nitration in m.3243A>G is probably involved in the mechanism related to the L-ARG supplementation benefits in MELAS patients.

## Data Availability

The data are presented within the article.

## References

[1] Böger R.H., Bode-Böger S.M. (2001). The clinical pharmacology of L-arginine. Annu. Rev. Pharmacol. Toxicol..

[2] McKnight J.R., Satterfield M.C., Jobgen W.S., Smith S.B., Spencer T.E., Meininger C.J. (2010). Beneficial effects of L-arginine on reducing obesity: Potential mechanisms and important impli-cations for human health. Amino. Acids.

[3] Stancic A., Filipovic M., Ivanovic-Burmazovic I., Masovic S., Jankovic A., Otasevic V., Korac A., Buzadzic B., Korac B. (2017). Early energy metabolism-related molecular events in skeletal muscle of diabetic rats: The effects of l -arginine and SOD mimic. Chem. Interactions.

[4] Vasilijevic A., Vojčić L., Dinulovic I., Buzadzic B., Korać A., Petrovic V., Jankovic A., Korac B. (2010). Expression pattern of thermogenesis-related factors in interscapular brown adipose tissue of alloxan-treated rats: Beneficial effect of l-arginine. Nitric. Oxide.

[5] Desquiret-Dumas V., Gueguen N., Barth M., Chevrollier A., Hancock S., Wallace D.C. (2012). Metabolically induced heteroplasmy shifting and l-arginine treatment reduce the energet-ic defect in a neuronal-like model of MELAS. Biochim. Biophys. Acta.

[6] El-Hattab A.W., Emrick L.T., Craigen W.J., Scaglia F. (2012). Citrulline and arginine utility in treating nitric oxide deficiency in mitochondrial disorders. Mol. Genet. Metab..

[7] Fryer R.H., Bain J.M., De Vivo D.C. (2016). Mitochondrial Encephalomyopathy Lactic Acidosis and Stroke-Like Epi-sodes (MELAS): A Case Report and Critical Reappraisal of Treatment Options. Pediatr. Neurol..

[8] El-Hattab A.W., Hsu J.W., Emrick L.T., Wong LJ C., Craigen W.J., Jahoor F., Scaglia F. (2012). Restoration of impaired nitric oxide production in MELAS syndrome with citrulline and argi-nine supplementation. Mol. Genet. Metab..

[9] Koga Y., Akita Y., Junko N., Yatsuga S., Povalko N., Fukiyama R., Ishii M., Matsuishi T. (2006). Endothelial dysfunction in MELAS improved by l-arginine supplementation. Neurol..

[10] Koga Y., Povalko N., Nishioka J., Katayama K., Yatsuga S., Matsuishi T. (2012). Molecular pathology of MELAS and l-arginine effects. Biochim. Biophys. Acta Gen. Subj..

[11] Koga Y., Ishibashi M., Ueki I., Yatsuga S., Fukiyama R., Akita Y., Matsuishi T. (2002). Effects of L-arginine on the acute phase of strokes in three patients with MELAS. Neurology.

[12] El-Hattab A.W., Emrick L.T., Williamson K.C., Craigen W.J., Scaglia F. (2013). The effect of citrulline and arginine supplementation on lactic acidemia in MELAS syndrome. Meta Gene..

[13] Coman D.J., Yaplito-Lee J., Boneh A. (2008). New indications and controversies in arginine therapy. Clin. Nutr..

[14] Litvinova L., Atochin D.N., Efattakhov N., Evasilenko M., Ezatolokin P., Ekirienkova E. (2015). Nitric oxide and mitochondria in metabolic syndrome. Front. Physiol..

[15] Elms S., Chen F., Wang Y., Qian J., Askari B., Yu Y., Pandey D., Iddings J.A., Caldwell R.B., Fulton D.J.R. (2013). Insights into the arginine paradox: Evidence against the importance of subcellular location of arginase and eNOS. Am. J. Physiol. Circ. Physiol..

[16] Popolo A., Adesso S., Pinto A., Autore G., Marzocco S. (2014). l-Arginine and its metabolites in kidney and cardiovascular disease. Amino. Acids.

[17] Rath M., Müller I., Kropf P., Closs E.I., Munder M. (2014). Metabolism via Arginase or Nitric Oxide Synthase: Two Competing Arginine Pathways in Macro-phages. Front. Immunol..

[18] Ghafourifar P., Asbury M.L., Joshi S.S., Kincaid E.D. (2005). Determination of Mitochondrial Nitric Oxide Synthase Activity. Methods Enzymol..

[19] El-Hattab A.W., Zarante A.M., Almannai M., Scaglia F. (2017). Therapies for mitochondrial diseases and current clinical trials. Mol. Genet. Metab..

[20] Bossy-Wetzel E., A Lipton S. (2003). Nitric oxide signaling regulates mitochondrial number and function. Cell Death Differ..

[21] King M.P., Koga Y., Davidson M., Schon E.A. (1992). Defects in mitochondrial protein synthesis and respiratory chain activity segregate with the tRNA(Leu(UUR)) mutation associated with mitochondrial myopathy, encephalopathy, lactic acidosis, and strokelike epi-sodes. Mol. Cell Biol..

[22] Gamba J., Gamba L.T., Rodrigues G.S., Kiyomoto B.H., Moraes C.T., Tengan C.H. (2012). Nitric Oxide Synthesis Is Increased in Cybrid Cells with m.3243A>G Mutation. Int. J. Mol. Sci..

[23] Barrientos A. (2002). In vivo and in organello assessment of OXPHOS activities. Methods.

[24] Venegas V., Wang J., Dimmock D., Wong L.-J.C. (2011). Real-Time Quantitative PCR Analysis of Mitochondrial DNA Content. Curr. Protoc. Hum. Genet..

[25] Ribeiro L., Silva F.D.A.E., Kurihara R.S., Schor N., Higa E.M. (2004). Evaluation of the nitric oxide production in rat renal artery smooth muscle cells culture exposed to radiocontrast agents. Kidney Int..

[26] Parikh S., Goldstein A., Koenig M.K., Scaglia F., Enns G.M., Saneto R., Anselm I., Cohen B.H., Falk M.J., Greene C. (2015). Diagnosis and management of mitochondrial disease: A consensus statement from the Mitochondrial Medicine Society. Genet. Med..

[27] Koenig M.K., Emrick L., Karaa A., Korson M., Scaglia F., Parikh S., Goldstein A. (2016). Recommendations for the Management of Strokelike Episodes in Patients With Mitochondrial Encephalomyopathy, Lactic Acidosis, and Strokelike Episodes. JAMA Neurol..

[28] Bombicino S.S., Iglesias D.E., Zaobornyj T., Boveris A., Valdez L.B. (2016). Mitochondrial nitric oxide production supported by reverse electron transfer. Arch. Biochem. Biophys..

[29] Tengan C.H., Rodrigues G.S., Godinho R.O. (2012). Nitric Oxide in Skeletal Muscle: Role on Mitochondrial Biogenesis and Function. Int. J. Mol. Sci..

[30] Koga Y., Akita Y., Nishioka J., Yatsuga S., Povalko N., Katayama K., Matsuishi T. (2007). MELAS and l-arginine therapy. Mitochondrion.

[31] Vattemi G., Mechref Y., Marini M., Tonin P., Minuz P., Grigoli L., Guglielmi V., Klouckova I., Chiamulera C., Meneguzzi A. (2011). Increased Protein Nitration in Mitochondrial Diseases: Evidence for Vessel Wall Involvement. Mol. Cell. Proteom..

[32] Xiong Y., Fru M.F., Yu Y., Montani J.-P., Ming X.-F., Yang Z. (2014). Long term exposure to L-arginine accelerates endothelial cell senescence through arginase-II and S6K1 signaling. Aging.

[33] MacPherson H., Noble B., Ralston S. (1999). Expression and functional role of nitric oxide synthase isoforms in human osteoblast-like cells. Bone.

[34] Rochette L., Lorin J., Zeller M., Guilland J.C., Lorgis L., Cottin Y., Vergely C. (2013). Nitric oxide synthase inhibition and oxidative stress in cardiovascular diseases: Possible therapeu-tic targets?. Pharmacol. Ther..

[35] Alkaitis M.S., Crabtree M.J. (2012). Recoupling the cardiac nitric oxide synthases: Tetrahydrobiopterin synthesis and re-cycling. Curr. Heart Fail Rep..

[36] Kobayashi E., Masuda M., Nakayama R., Ichikawa H., Satow R., Shitashige M. (2010). Reduced argininosuccinate synthetase is a predictive biomarker for the development of pulmo-nary metastasis in patients with osteosarcoma. Mol. Cancer Ther..

[37] Scalera F., Closs E.I., Flick E., Martens-Lobenhoffer J., Boissel J.P., Lendeckel U., Heimburg A., Bode-Böger S.M. (2009). Paradoxical effect of l-arginine: Acceleration of endothelial cell senescence. Biochem. Biophys. Res. Commun..

[38] Li H., Meininger C.J., Hawker J.R., Haynes T.E., Kepka-Lenhart D., Mistry S.K., Morris S.M., Wu G. (2001). Regulatory role of arginase I and II in nitric oxide, polyamine, and proline syntheses in endothelial cells. Am. J. Physiol. Metab..

[39] Moretto J., Guglielmetti A.-S., Tournier-Nappey M., Martin H., Prigent-Tessier A., Marie C., Demougeot C. (2017). Effects of a chronic l -arginine supplementation on the arginase pathway in aged rats. Exp. Gerontol..

